# Dairy product intake and mortality in a cohort of 70-year-old Swedes: a contribution to the Nordic diet discussion

**DOI:** 10.1007/s00394-017-1556-2

**Published:** 2017-10-28

**Authors:** Gianluca Tognon, Elisabet Rothenberg, Martina Petrolo, Valter Sundh, Lauren Lissner

**Affiliations:** 1 Section for Epidemiology and Social Medicine (EPSO), Department of Public Health and Community Medicine, Sahlgrenska Academy, University of Gothenburg, Box 453, SE 405 30 Gothenburg, Sweden; 2 Food and Meal Science, Kristianstad University, Kristianstad, Sweden

**Keywords:** Elderly, Aged, Nordic diet, Diet quality, Dairy products, Cheese, Mortality

## Abstract

**Introduction:**

Conflicting results in the literature exist on the role of dairy products in the context of a Nordic Healthy Diet (NHD). Two recent Swedish studies indicate both negative and positive associations with total mortality when comparing key dairy products. There is no consensus about how to include these foods into the NHD.

**Purpose:**

To study consumption of cheese and milk products (milk, sour milk and unsweetened yoghurt) by 70-year-old Swedes in relation to all-cause mortality.

**Methods:**

Cox proportional hazard models, adjusted for potential confounders and stratified by follow-up duration, were used to assess the prediction of all-cause mortality by the above foods. The associations of fat from cheese and milk products with mortality were tested in separate models.

**Results:**

Cheese intake inversely predicted total mortality, particularly at high protein intakes, and this association decreased in strength with increasing follow-up time. Milk products predicted increased mortality with stable HRs over follow-up. The association between milk products and mortality was strongly influenced by the group with the highest consumption. Fat from cheese mirrored the protective association of cheese intake with mortality, whereas fat from milk products predicted excess mortality, but only in an energy-adjusted model.

**Conclusion:**

Based on our results, it may be argued that the role of dairy products in the context of a Nordic healthy diet should be more clearly defined by disaggregating cheese and milk products and not necessarily focusing on dairy fat content. Future epidemiological research should consider dairy products as disaggregated food items due to their great diversity in health properties.

**Electronic supplementary material:**

The online version of this article (doi:10.1007/s00394-017-1556-2) contains supplementary material, which is available to authorized users.

## Introduction

Nordic nutrition researchers have recently defined a new healthy diet, which could possess the same health properties as of the well-known Mediterranean diet, but based on typical Nordic foods that are frequently consumed by the Nordic population [1]. The positive health effects of this diet have been attributed to foods that can be locally produced in the Nordic countries (e.g. apples, pears and berries, root vegetables and cabbages, wholegrain oats and rye, salmon and herring as well as boiled potatoes), thereby excluding typical Nordic foods with potential harmful effects such as fresh and processed meat. Notably, dairy products, which were usually considered unhealthy in the Mediterranean diet score [2, 3], have often been excluded from the definition of a healthy Nordic diet [4–6], or considered only if low in fat [7–9], in line with the Nordic Dietary Recommendations [1]. The ambiguous role of dairy products in the definition of the healthy Nordic diet may relate to the fact that their health properties have long been a sensitive topic in Sweden and elsewhere. Indeed, the promotion of the health properties of dairy products has a long history in Sweden; a milk propaganda campaign many decades ago was particularly successful in making Swedes become among the greatest per capita milk consumers worldwide [10].

Recent evidence from two large population studies in Sweden re-opened the discussion about the health effects of dairy products by demonstrating that higher milk intakes were positively associated with all-cause mortality, whereas cheese appeared to be protective [11, 12]. However, a recent meta-analysis summarizing the evidence about the association between intake of milk and milk products on mortality showed an overall neutral effect of dairy products [13].

Considering the above premises, and based on prospective population studies of men and women living in Gothenburg [14–19] who underwent diet history interviews at the age of 70, our goal was to determine whether cheese intake was associated with mortality in a different way, compared to milk products.

## Subjects and methods

### Study population

This study is based on the Gerontological and Geriatric Population Studies in Gothenburg (H70) a prospective cohort study that recruited 1381 men and women aged 70 and born in 1901, 1911, 1922 and 1930 [14, 15, 17–20]. Women belonging to the two latest-born cohorts were jointly examined with the Prospective Population Study of Women in Gothenburg (PPSW) [16]. In both studies, subjects were sampled based on the day of birth, and invited to participate. Response rate decreased with time, from 84 and 86% in the earliest birth cohort to 65% in the most recent birth cohorts with general similar rates in men and women in most cohorts.

These cohorts have been monitored continuously for mortality by linking personal identification numbers with the national death registration system. The present study is based on the mortality follow-up on May 21, 2010 with a mean follow-up of 13.2 years (max follow-up duration = 43 years).

For subjects with incomplete information on key covariates, decisions on exclusions were based on analyses of mortality (not shown). Subjects with missing BMI (*n* = 165) were excluded because of increased mortality risk compared to rest of the population. Since this was not the case for subjects with missing education (*n* = 39) and physical activity (*n* = 13), dummy variables were added to the models to retain these subjects into the analyses. For subjects with missing marital status (*n* = 3) a dummy variable could not be created because of the small number and these subjects were therefore not retained in fully adjusted models. The final study sample included 1213 subjects (678 females) of whom 833 died of any cause during the observation period (431 females).

### Dietary assessment

All subjects in the prevent analysis had diet history data (described below) from 1971, 1981, 1992 or 2000. This method, which has been extensively described elsewhere, provided a detailed assessment of usual intakes of a large number of food items and mixed dishes [14, 17–19]. The dietary assessment was previously validated by comparison of energy intake with estimated total energy expenditure (TEE) by heart rate monitoring, activity diary and double labelled water as well as by calculating the ratio between energy intake and basal metabolic rate (BMR) [17, 19, 21]. The dietary assessment consisted of individual interviews by trained dieticians, who enquired about the intakes of food items through a multi-pass process that ensured a high level of completeness. Therefore missing values were reasonably interpreted as null intakes and set to 0 g/day.

Two dietary variables were generated from the sum of either cheese intakes (all types) or a group of dairy products including milk, soured milk and unsweetened yoghurt, hereby referred as “milk products” for simplicity. Although the latter items appeared in all surveys, they could not be analyzed separately since they were combined differently in the various cohorts, but we were able to harmonize them into a single food group for this paper. Fat intakes from both food groups were also calculated. Total protein intake was divided by body weight and dichotomized based on a cut-off of 1.2 g/kg body weight/day [22].

### Covariates measured at baseline

Body weight and height were measured by trained staff on the day of the physical examination as described previously [15, 23] and BMI (weight/height^2^) was calculated. Physical activity was assessed by asking subjects about their leisure time physical activity during adult life and levels were dichotomized into “physically active” and “physically inactive” [23]. Information about smoking history, education, and current marital status were obtained for all subjects.

### Statistical analyses

Continuous variables representing either cheese or milk product intakes were tested in relation to their associations with all-cause mortality in Cox proportional hazard models. In the latter, the follow-up time was included as number of days after the baseline examination. These models were either adjusted for sex and birth cohort only or adjusted for the following covariates: birth cohort, sex, marital status (married/not married), BMI, smoking status (ever vs never smokers), education (basic vs higher levels) and physical activity level (low activity vs higher). In complementary analyses, high protein intakes (> 1.2 g/kg body weight), alcohol intake and fat intake were separately included in the models.

Since assessment of the risk associated with an increased intake of 1 g per day would have generated very small HRs, intakes were divided by either 10 (cheese) or 100 (milk products) in order to increase the interpretability of hazard ratios. In terms of portions, 10 g correspond to the average single portion of cheese consumed in Sweden, where it is common to cut cheese in thin slices, whereas 100 g of milk products corresponds to approximately 1/5 of a pint, a measure commonly referred to in previous studies [13]. The Cox models were run stratifying by birth cohort by means of the STRATA command available in PROC PHREG (SAS 9.4). This allows the baseline risk for each birth cohort to vary, taking into consideration potential differences in total mortality risk among birth periods without reducing the power of the statistical analyses. Because the assumption of proportional hazards was not fulfilled in this study, we chose to present the results stratified by duration of follow up. Additionally, in order to assess whether the use of dummy variables to retain subjects with missing information regarding potential confounders could have altered our results, we repeated our main analyses using multiple imputation [24] which estimated missing values for education and physical activity. To assess whether the presence of subclinical or pre-existing conditions at the time of the recruitment was likely to have influenced the above-mentioned analyses, we repeated the latter by excluding the subjects who died during the first 2 years of follow-up.

In addition to the analysis of continuous intakes, dose–response was tested by comparing the first sex-specific tertile of cheese and milk product intakes with the second and third tertiles, in Cox models adjusted for the above-mentioned covariates. Also, to further analyze potential non-linear relations between the intakes and mortality, we compared the likelihoods of three different models: (1) those with cheese and milk product intakes included as continuous variables, (2) those also including a quadratic term (i.e. squared cheese intake or squared milk product intakes), and (3) those using piecewise regression models [25] to produce separate HRs on four intervals of intakes. An interaction between cheese and milk products with respect to mortality was tested to determine whether the effect of one dairy product might be modified by the other. A likelihood ratio test comparing model chi square values assessed whether the difference in the overall model chi square (-2Log Likelihood) of two models (e.g. including an extra factor such as milk in the cheese models or an interaction factor between cheese and milk) is significant, assuming that the model with the highest likelihood gives the best prediction of mortality. This test is asymptotically the same as the result reported from the Wald test (where two variables plus their product are included in the same model), but is considered more reliable when the sample size is limited. By applying the same procedure, we tested effect modification by alcohol intake on the association of either cheese or milk product intakes on total mortality. In order to assess whether the participants’ nutritional status could have modified the association between dairy intake and mortality, we tested effect modification by BMI and high protein intake (dichotomized using 1.2 g/kg body weight as threshold [22]) on the association of these food groups with mortality.

Fat intakes from either cheese or milk products were tested against mortality in Cox models adjusted for the above-mentioned covariates plus total fat intake. Analyses have been performed in SAS 9.4 with the exception of multiple imputation [24] which has been done in Stata 13.

### Bioethics

All examinations since 1992 were approved by the Gothenburg University Ethics Committee. In accordance with the Declaration of Helsinki (1989) of the World Medical Association, all participants were informed of the aims and procedures of the study and gave their consent (Ethical approval no. 179–92 and no. Ö 402–99).

## Results

### Descriptive analyses

Covariates included in the analyses are described after stratification by birth cohort (Table 1). Mean BMI tended to increase across birth cohorts (from 25.6 ± 3.6 to 27.0 ± 4.0 kg/m^2^) whereas a slight decrease in mean fat intake was observed over time. The percent of subjects with an education above the basic level in the latest birth cohort (40.1%) was more than doubled compared to the earliest one (17.6%). Again, comparing earlier vs. latest born cohorts, subjects who never smoked decreased from 50.3 to 45.8%. Finally, the prevalence of alcohol use was 57.4% in the earliest birth cohort and 87.9% in the latest one. Since no interaction was found between sex and the two dietary exposure variables, the descriptive data in Table 1 is aggregated by sex. Daily intakes of each food group included in the score calculations are depicted in Supplementary Table 1, stratified by both sex and birth cohort.

**Table 1:** (new): Descriptive analyses of the sample study stratified by birth cohorts. *p* for trends were calculated from unadjusted linear models. SD = Standard Deviation

	1901 **(** ***n*** = **324)**	1911 **(** ***n*** = **232)**	1922 **(** ***n*** = **164)**	1930 **(** ***n*** = **496)**	*p* for trend
Women/men	158/166	115/117	116/48	291/205	< 0.001
Follow-up years to mortality (mean ± SD)	12.9 ± 7.4	13.4 ± 7.1	15.8 ± 6.2	12.7 ± 3.0	0.98
BMI (kg/m^2^, mean ± SD)	25.6 ± 3.6	26.5 ± 4.1	26.1 ± 3.8	27.0 ± 4.0	< 0.0001
Total energy intake (kcal, mean ± SD)	2074.5 ± 464.0	2195.3 ± 513.1	2021.9 ± 465.0	2145.7 ± 521.0	0.35
Fat intake (%, mean ± SD)	36.5 ± 5.1	37.5 ± 5.1	35.3 ± 6.0	34.9 ± 6.0	< 0.0001
Protein intake (%, mean ± SD)	3.6 ± 0.5	3.5 ± 0.6	4.1 ± 0.7	4.0 ± 0.6	< 0.0001
Alcohol users (%)	57.4	70.7	80.5	87.9	< 0.0001
Alcohol intake among users (mean ± SD)	5.3 ± 4.7	8.3 ± 11.4	6.4 ± 9.7	9.4 ± 12.1	< 0.0001
Cheese intake (g/day, mean ± SD)	31.4 ± 20.3	33.6 ± 23.8	44.5 ± 34.6	38.9 ± 26.5	< 0.01
Fat from cheese (g/day, mean ± SD)	10.3 ± 5.8	10.6 ± 7.2	11.6 ± 9.0	11.6 ± 9.6	0.02
Milk product intake (g/day, mean ± SD)	369.9 ± 236.9	437.9 ± 240.5	347.0 ± 219.6	341.8 ± 264.6	< 0.001
Fat from milk products (g/day, Mean ± SD)	8.2 ± 6.8	9.4 ± 6.7	5.1 ± 4.2	5.1 ± 5.3	< 0.0001
Low physical activity (%)	14.5	23.3	16.5	7.7	< 0.0001
Education above basic level (%)	17.6	24.1	32.9	40.1	< 0.0001
Never smokers (%)	50.3	45.3	47.6	45.8	0.25
Married (%)	62.7	65.1	46.3	60.1	0.3

### Cheese and milk product intakes and all-cause mortality

Table 2 shows the association between intakes of cheese and milk products in relation to all-cause mortality, in either crude or fully adjusted models stratified by the duration of follow up. Cheese intake was inversely associated with mortality and the strength of this association tended to decrease with longer follow-up times. On the other hand, milk product intakes showed a positive association with mortality that did not seem to be influenced by follow-up duration. These results were confirmed both by excluding the first 2 years of follow-up and by estimating missing values for education and physical activity by multiple imputation.

**Table 2:** Association between cheese intake (10 g/day) and milk products (100 g/day) with all-cause mortality, assessed in a Cox regression proportional hazard models both for the total follow-up duration and stratified by duration of follow-up

Follow-up duration	Cases	Cheese		Other dairy products	
		Basic model^1^ HRs (95% confidence limits)	Adjusted model^2^ HRs (95% confidence limits)	Basic model^1^ HRs (95% confidence limits)	Adjusted model^2^ HRs (95% confidence limits)
12 years	411	0.93 (0.90; 0.97)^***^	0.94 (0.91; 0.98)^**^	1.04 (1.00; 1.08)	1.04 (1.00; 1.08)
20 years	728	0.96 (0.94; 0.99)^**^	0.97 (0.94; 1.00)^*^	1.04 (1.00; 1.07)^*^	1.04 (1.01; 1.08)^*^
32 years	831	0.96 (0.94; 0.98)^**^	0.97 (0.94; 0.99)^*^	1.03 (1.00; 1.07)^*^	1.04 (1.01; 1.08)^**^
Total	833	0.91 (0.86; 0.97)^**^	0.92 (0.87; 0.98)^**^	1.06 (1.00; 1.13)^*^	1.06 (1.00; 1.12)^‡^

The analyses on the total follow-up duration were obtained from models including an interaction term between exposure and follow-up time

^*^ *p* value < 0.05, ^**^ *p* value < 0.01, ^***^ *p* value < 0.001, ^‡^ *p* = 0.07

^1^Adjusted for sex and birth cohort (included as a stratification variable)

^2^Adjusted for sex, birth cohort (included as a stratification variable), smoking status, BMI, education, marital status, physical activity and total energy intake

Table 3 shows associations across three tertiles of cheese and milk product intakes at different follow up times, adjusted for the above confounders. The association between cheese and mortality was stable across tertiles, whereas a tendency for a dose–response effect was observed for milk products, particularly at the longest follow-up.

**Table 3:** Association between intakes of cheese and milk products with all-cause mortality, across increasing sex-specific tertiles of intakes and at different follow-up durations, assessed in Cox proportional hazard models

Follow-up duration	Cases/tot. subjects	HR (95% confidence intervals)^1^			*p* for trend
		Cheese			
		Low *M* 0–28.7 *F* 0–21.4	Med *M* 3.0–45.0 *F* 21.5–44.5	High *M* > 45.0 *F* > 45.0	
12 years	411/1213	1	0.87 (0.69; 1.09)	0.80 (0.61; 1.03)	n.s
20 years	728/1213		0.92 (0.77; 1.10)	0.92 (0.76; 1.12)	n.s
32 years	831/1213		0.92 (0.78; 1.08)	0.89 (0.74; 1.07)	n.s
Total	833/1213		0.82 (0.65; 1.03)	0.71 (0.48; 1.04)	n.s

Follow-up duration	Cases/tot. subjects	Other dairy products			*p* for trend
		Low *M* 0–286 *F* 0–210	Med *M* 290–475 *F* 214–400	High *M* > 500 *F* > 403	
12 years	411/1213	1	1.03 (0.80; 1.32)	1.08 (0.84; 1.39)	n.s
20 years	728/1213		0.98 (0.82; 1.19)	1.17 (0.97; 1.42)	n.s
32 years	831/1213		1.09 (0.91; 1.29)	1.20* (1.00; 1.44)	< 0.05
Total	833/1213		1.04 (0.81; 1.32)	1.09 (0.75; 1.59)	n.s

Tertile cut-offs are reported in g/day for both males (*M*) and females (*F*). The analyses on the total follow-up duration were obtained from models including an interaction term between exposure and follow-up time

*n.s*. not significant

*p value < 0.05

^1^Adjusted for sex, birth cohort (included as a stratification variable), smoking status, BMI, education, marital status, physical activity and total energy intake

When testing non-linearity in piecewise models, we found that cheese intake had an almost constant inverse association with mortality across intake levels, whereas the association of milk products with mortality was mainly driven by the highest intake levels, with the most marked increase in mortality among those drinking > 800 g/day of milk products. These subjects were mostly sedentary, unmarried men, with higher BMI and high energy intake. Similar results were not found for cheese. Using the likelihood ratio tests, we compared the piecewise model with two models which included either cheese and milk product intakes as continuous variables, or quadratic terms of the intake variables. The likelihood ratio tests were all non-significant.

No interaction between cheese and milk product intakes in relation to their association with mortality was found by likelihood ratio test. The two variables showed a very low correlation with each other (*r* = 0.04) and, when they were included in the same model, their associations with mortality remained unchanged. Associations with mortality of cheese and milk products were not modified by alcohol intake.

A high protein intake was found to modify the association between cheese intake (10 g/day) and all-cause mortality (*p* for interaction < 0.05) in a way that the latter association was statistically significant at higher (HR = 0.92, 95% CI 0.88; 0.96, *p* < 0.0001) but not at lower protein intakes (HR = 1.00, 95% CI 0.97; 1.05). No evidence for a similar effect modification was found for milk products. Protein intake was positively correlated with BMI (*r* = 0.10, *p* < 0.001).

### Dairy fat intake and all-cause mortality

Fat intake from cheese was inversely associated with all-cause mortality (HR = 0.86, 95% CI 0.78; 0.95). This association did not materially change when the model was adjusted for either total fat or total energy intake. Fat intake from milk products showed a non-statistically significant positive association (HR = 1.09, 95% CI 0.97; 1.22), which became significant after adjustment by either total fat or energy intake (HR = 1.18, 95% CI 1.04; 1.34). Neither type of fat was related to BMI (data not shown).

## Discussion

The present paper investigated the association of cheese and milk products (i.e. fermented and non-fermented milk) with all-cause mortality and found that cheese intake was negatively associated with mortality, in line with previous results from other two large Swedish studies [11, 12]. In the present study, the association between cheese and mortality did not show any dose–response and maintained statistical significance despite somewhat decreased strength along the follow-up. In addition, this association seemed to be modified by high protein intake, but not by weight status. A progressive decrease in strength along a lengthy follow-up has already been observed in nutritional epidemiology for other types of associations, for instance in relation to serum vitamin D levels [26, 27] as well as in dietary exposures [28]. Such attenuation in prognostic value of single assessments over time may be attributed to changes in exposure during the follow-up [29].

Since the effects of dairy products on health have traditionally been linked to fat content, we also tested the association between fat from either cheese or milk products and mortality. The result was that the intake of fat from cheese was inversely associated with all-cause mortality, whereas the intake of fat from milk products showed a weak tendency toward a positive association. If dairy fat explained the results obtained here, we would have expected that the intakes of fat from cheese as well as fat from milk products to have similar associations with mortality. Instead, both types showed essentially the same association as the type of dairy they were contained. Therefore, no clear conclusion could be drawn regarding the role of fat from milk products which seemed to be more related to total energy intake. Notably, in our recent study [12], which was based on a larger adult Swedish population, the intakes of low-, medium- and high-fat non-fermented milk were all positively associated with mortality, although high-fat milk intake showed the highest HR.

A potential explanation of the opposite associations of both cheese and cheese fat intakes with mortality compared to milk products might involve the production of healthy bioactive compounds during milk fermentation [30], including some types of healthy saturated fats [31]. In line with this hypothesis, yoghurt consumption has been found to be associated with a healthy weight status and the prevention of type 2 diabetes [32, 33]. Cheese is a source of vitamin K2 (menaquinone) [34] which has been previously associated with a lower risk of cardiovascular disease [35] and which may have contributed to the reduction of mortality risk among cheese consumers. Finally, according to a recently proposed alternative hypothesis, milk fermentation might have health benefits through reducing galactose concentration in milk and the higher oxidation rate related to galactose intake [24].

Although we could not specifically compare fermented vs non-fermented milk, it is interesting to mention that National Statistics show that the per capita availability of non-fermented milk was predominant over fermented milk across the years when our study participants were interviewed (% of fermented milk between < 5% in 1960 to 21% in 2000) [36]. Therefore, we can prudently assume that the results we obtained for combined milk, sour milk and yoghurt could be attributed mostly to milk intake, and that the hazard ratios related to milk intake might have been diluted by a growing availability of fermented milk during the latest recruitment period.

The controversy surrounding health effects of dairy products is not new. An example of this is the evidence for a protective effect of dairy product intake on colon and bowel cancer that contrasts with the positive (although weak) association with prostate cancer [37]. However, there are reasons to believe that the role of dairy intake in elderly Swedes born between 1901 and 1930 may be particularly relevant to investigate. In the period between the 1930s and 1950s, the Swedes had high milk intakes, as a consequence of a previously mentioned campaign approved by doctors, teachers, public health authorities and other experts, and aimed to promote the health effects of milk. In particular, the lobby organization “Mjölkpropagandan” (Milk propaganda), founded in 1923, is probably one of the main reasons why many Swedes (and particularly our study participants, who were young at that time) still view milk as a very healthy food and a good source of nutrients. Milk was often assumed to be an effective way to prevent undernourishment, which had a high prevalence at the time when many of the subjects belonging to this study were born [10].

In our previous paper about the Mediterranean diet in the same population studied here, all dairy products were aggregated and scored as unhealthy foods. In that analysis they were positively associated with mortality, in contrast to the present analyses which highlighted a potentially opposite association between cheese and milk products in relation to all-cause mortality. The present results show the need to disaggregate cheese from milk products when testing the association between the Mediterranean Diet Score, the Healthy Nordic Diet score, and other a priori dietary scores used in epidemiological studies. In particular, since dairy is a typical Nordic food group, the definition of the Nordic Healthy Diet should take into consideration the role of dairy type as well as dairy fat content. Also, it is interesting that the association between milk and mortality, which has been tested in several studies from around the world [13], has only been observed in two big Swedish studies [11, 12]. However, the opposite associations found for cheese and milk products in relation to mortality, may have relevance beyond Sweden.

Our study has both strengths and limitations, the former being the high quality of nutritional data obtained by a diet history during a face to face multi-pass interview with the dietician, validated by the high EI/BMR ratio [17, 19]. Also, although the results cannot be generalized to the whole population, we believe that the initially high response rates (in both men and women) [15] and the homogeneous age at baseline allow us to consider our results generalizable at least to the Swedish population aged 70. The decline in participation rates in this study has been discussed by Eiben et al. [14], who tested whether non-participants differed in any measurable way from participants. Specifically, similar values for self-rated health, history of myocardial infarction, smoking status and diabetes incidence were found, although unmarried men were significantly under-represented.

The limitations of this study include lack of repeated dietary assessments, small sample size and the fact that milk products could not be analysed separately. It is also worth mentioning that, although the analyses were always adjusted for birth cohort, the results could still be influenced by a residual cohort effect, as the examinations spanned over a large range of time during which living conditions changed in numerous ways. However, when comparing subjects who were born in Sweden in 1901 vs those born 1930, the Swedish National Bureau of Statistics reports that life expectancy at 65 years of age did not differ greatly [38]. Rather, it is the life expectancy at birth which has increased substantially, due to the decrease in infant infections and mortality at early ages, which presumably could not have influenced the results of this study.

We conclude that milk and cheese products complicate the application of a Nordic healthy diet concept in epidemiology. Future studies should address the role of cheese and milk product intake in relation to longevity not only in older adults, but also across the life course.

## Electronic supplementary material

Below is the link to the electronic supplementary material.

Supplementary material 1 (DOCX 27 KB)
