# Supplementary material for: Dairy product intake and mortality in a cohort of 70-year-old Swedes: a contribution to the Nordic diet discussion
Source: Eur J Nutr. 2017 Oct 28;57(8):2869–76. doi: 10.1007/s00394-017-1556-2 (PMC6267406; doi:10.1007/s00394-017-1556-2)
Supplement: Supplementary file 1 — Supplementary material 1 (DOCX 27 KB) [file 394_2017_1556_MOESM1_ESM.docx]

**SUPPLEMENTARY TABLE**

|  | **Medians (25-75^th^ pct) (g/day)** | | | | | | | |
| --- | --- | --- | --- | --- | --- | --- | --- | --- |
| **Food group** | **Females** | | | | **Males** | | | |
|  | **1901**  **(n = 187)** | **1911**  **(n = 130)** | **1922**  **(n = 136)** | **1930**  **(n = 320)** | **1901**  **(n = 182)** | **1911**  **(n = 131)** | **1922**  **(n = 63)** | **1930**  **(n = 232)** |
| Tot. vegetables | **43.7**  (*22.8; 75.0*) | **44.0**  (*24.5; 77.5*) | **117.3**  (*65.7; 184.0*) | **125.0**  (*74.2; 187.4*) | **38.3**  (*16.2; 63.0*) | **35.0**  (*15.0; 54.0*) | **86.6**  (*48.9; 130.0*) | **96.7**  (*62.5; 158.2*) |
| Root veget. & Cabbages | **15.0**  (*9.3; 29.3*) | **12.0**  (*5.0; 25.0*) | **53.1**  (*26.1; 87.2*) | **47.9**  (*23.4; 87.1*) | **13.3**  (*8.0; 25.0*) | **12.0**  (*7.0; 24.0*) | **54.3**  (*28.9; 85.4*) | **39.0**  (*16.4; 77.9*) |
| Total fruit | **118.2**  (*78.8; 210.6*) | **120.4**  (*65.4; 173.6*) | **143.0**  (*97.3; 242.5*) | **185.1**  (*115.4; 271.2*) | **122.5**  (*57.3; 203.7*) | **106.9**  (*51.0; 188.0*) | **122.9**  (*68.0; 205.8*) | **154.3**  (*88.9; 228.3*) |
| Nordic fruit  (apples, berries, pears) | **55.6**  (*18.2; 105.0*) | **54.3**  (*19.3; 105.6*) | **58.6**  (*38.8; 116.5*) | **97.6**  (*45.0; 134.6*) | **52.5**  (*20.1; 105.0*) | **44.8**  (*16.4; 105.6*) | **51.2**  (*31.1; 92.7*) | **75.0**  (*30.0; 108.0*) |
| Boiled potatoes | **92.1**  (*66.3; 121.6*) | **91.8**  (*58.7; 128.4*) | **69.3**  (*44.4; 101.8*) | **68.6**  (*45.0; 100.0*) | **138.1**  (*93.9; 184.2*) | **147.1**  (*110.5; 206.6*) | **95.6**  (*59.4; 165.8*) | **101.3**  (*67.5; 143.0*) |
| Total fish | **32.4**  (*21.6; 43.2*) | **19.2**  (*10.7; 28.4*) | **28.8**  (*15.0; 46.3*) | **30.0**  (*20.0; 46.7*) | **40.5**  (*27.0; 54.0*) | **23.5**  (*16.2; 34.0*) | **26.7**  (*16.7; 50.0*) | **35.1**  (*20.0; 53.3*) |
| Nordic fish | **32.4**  (*21.6; 43.2*) | **19.2**  (*10.7; 28.4*) | **26.3**  (*14.1; 46.3*) | **29.2**  (*20.0; 46.4*) | **40.5**  (*27.0; 54.0*) | **23.5**  (*16.2; 34.0*) | **26.7**  (*15.2; 50.0*) | **33.3**  (*20.0; 53.3*) |
| Wholegrain cereals | **39.7**  (*17.0; 86.7*) | **82.5**  (*50.0; 130.0*) | **72.0**  (*50.6; 105.0*) | **73.8**  (*42.0; 108.1*) | **39.3**  (*20.0; 85.0*) | **58.5**  (*24.0; 109.0*) | **105.0**  (*63.5; 149.3*) | **90.0**  (*58.0; 133.9*) |
| Dairy products |  |  |  |  |  |  |  |  |
| Cheese | **30.0**  (15.0; 45.0) | **30.0**  (15.0; 45.0) | **33.7**  (20.0; 60.0) | **39.0**  (20.0; 60.0) | **30.0**  (30.0; 45.0) | **30.0**  (15.0; 45.0) | **45.0**  (30.0; 63.0) | **46.9**  (28.5; 80.0) |
| Milk, soured milk, unsweetened yoghurt | **300.0**  (200.0; 500.0) | **400.0**  (300.0; 500.0) | **300.0**  (200.0; 440.0) | **286.0**  (171.4; 428.6) | **400.0**  (300.0; 500.0) | **500.0**  (300.0; 600.0) | **400.0**  (250.0; 500.0) | **350.0**  (200.0; 500.0) |
| Low-fat dairy products | **300.0**  (*200.0; 450.0*) | **350.0**  (*200.0; 500.0*) | **300.0**  (*160.0; 404.8*) | **253.6**  (*142.9; 406.0*) | **400.0**  (*300.0; 500.0*) | **400.0**  (*200.0; 550.0*) | **401.3**  (*269.0; 506.4*) | **300.0**  (*150.0; 500.0*) |

**Supplementary table 1:** Intakes (medians and 25-75^th^ percentiles) of Nordic food items and commonly consumed dairy products stratified by sex and birth cohort.
